# Direct Pore Binding as a Mechanism for Isoflurane Inhibition of the Pentameric Ligand-gated Ion Channel ELIC

**DOI:** 10.1038/srep13833

**Published:** 2015-09-08

**Authors:** Qiang Chen, Monica N. Kinde, Palaniappa Arjunan, Marta M. Wells, Aina E. Cohen, Yan Xu, Pei Tang

**Affiliations:** 1 Department of Anesthesiology, University of Pittsburgh School of Medicine, Pittsburgh, Pennsylvania, 15260, USA; 2 Department of Pharmacology and Chemical Biology, University of Pittsburgh School of Medicine, Pittsburgh, Pennsylvania, 15260, USA; 3 Department of Structural Biology, University of Pittsburgh School of Medicine, Pittsburgh, Pennsylvania, 15260, USA; 4 Department of Computational and Systems Biology, University of Pittsburgh School of Medicine, Pittsburgh, Pennsylvania, 15260, USA; 5 Stanford Synchrotron Radiation Lightsource, 2575 Sand Hill Road, MS: 99, Menlo Park, California 94025, USA.

## Abstract

Pentameric ligand-gated ion channels (pLGICs) are targets of general anesthetics, but molecular mechanisms underlying anesthetic action remain debatable. We found that ELIC, a pLGIC from *Erwinia chrysanthemi,* can be functionally inhibited by isoflurane and other anesthetics. Structures of ELIC co-crystallized with isoflurane in the absence or presence of an agonist revealed double isoflurane occupancies inside the pore near T237(6′) and A244(13′). A pore-radius contraction near the extracellular entrance was observed upon isoflurane binding. Electrophysiology measurements with a single-point mutation at position 6′ or 13′ support the notion that binding at these sites renders isoflurane inhibition. Molecular dynamics simulations suggested that isoflurane binding was more stable in the resting than in a desensitized pore conformation. This study presents compelling evidence for a direct pore-binding mechanism of isoflurane inhibition, which has a general implication for inhibitory action of general anesthetics on pLGICs.

General anesthetics are administered to approximately 256 million people each year^1^. In addition to their primary action to render analgesia, amnesia, immobility, and unconsciousness, these drugs are also implicated in unwanted side effects, including post-operative cognitive decline^2^,^3^ and neurotoxicity in pediatric and elderly populations^4^,^5^,^6^. At the molecular level, how general anesthetics exert their on-target and off-target actions remains poorly understood.

Functional measurements suggest that a superfamily of pentameric ligand-gated ion channels (pLGICs) plays a central role in anesthetic action^7^,^8^. At clinically relevant concentrations, general anesthetics inhibit agonist-elicited currents of cation-conducting channels, such as nicotinic acetylcholine receptors (nAChRs), and potentiate currents of anion-conducting channels, such as GABA_A_ and glycine receptors^7^,^8^. These channels regulate a myriad of sensory processes and their malfunctions are directly linked to several neurological disorders. A structural insight into the mode of action, particularly the molecular details of anesthetic binding sites, is essential to understand the functional modulation of pLGICs by general anesthetics.

Discrete anesthetic binding sites have been proposed on the basis of various experimental and computational studies. In the extracellular domain (ECD), which contains the orthosteric agonist-binding site, crystal structures of prokaryotic pLGICs from *Gloeobacter violaceus* (GLIC) and *Erwinia chrysanthemi* (ELIC) have revealed binding sites for anesthetics: ketamine at the interface of two subunits in GLIC^9^ and bromoform at a similar location in ELIC^10^. Photoaffinity labeling^11^ and computational studies^12^,^13^,^14^ have also provided evidence for anesthetic binding in the ECD of nAChRs. Some of these reported ECD sites partially overlap with the agonist or antagonist binding sites^9^,^11^. In the transmembrane domain (TMD) of pLGICs, diverse intra- and inter-subunit allosteric sites for general anesthetic binding have been identified through various approaches, including NMR spectroscopy^15^,^16^,^17^, photoaffinity labeling^11^,^18^,^19^,^20^,^21^, crystallography^10^,^22^,^23^, and molecular dynamics (MD) simulations^12^,^13^,^14^,^24^,^25^. Specifically for the pore in the TMD, previous site-directed mutagenesis, electrophysiology measurements, and photoaffinity labeling suggested an open-channel blocking mechanism for functional inhibitions by anesthetics^26^,^27^,^28^,^29^,^30^, but available structural data were not sufficient to support such a mechanism^10^.

In this study, we co-crystalized ELIC with the general anesthetic isoflurane, which is known to inhibit mammalian neuronal and muscle nAChRs^31^,^32^ and GLIC^33^. The crystal structures of ELIC-isoflurane were determined with resolutions of 3.0 Å and 3.4 Å in the absence and presence of agonists, corresponding to the presumed resting and desensitized states of ELIC, respectively. These crystal structures revealed double isoflurane occupancies near the 6′ and 13′ positions inside the pore of ELIC. Site-directed mutagenesis and subsequent functional measurements further showed a direct pore-binding mechanism of isoflurane inhibition. In addition to the previously proposed open-channel blocking action^26^,^27^,^28^, the direct pore-binding mechanism suggested by our high-resolution structural data contains another component of anesthetic inhibition, which is achieved by stabilizing the resting channel conformation. Moreover, similar to isoflurane, several other representative general anesthetics, including halothane, sevoflurane, propofol, thiopental, and etomidate, also inhibit ELIC and do so as effectively on ELIC as on nAChRs. This study provides solid structural data to support the significance of direct pore binding as a mechanism of functional inhibition of pLGICs by general anesthetics.

## Results

### General anesthetics inhibit function of ELIC

**Figure 1:**
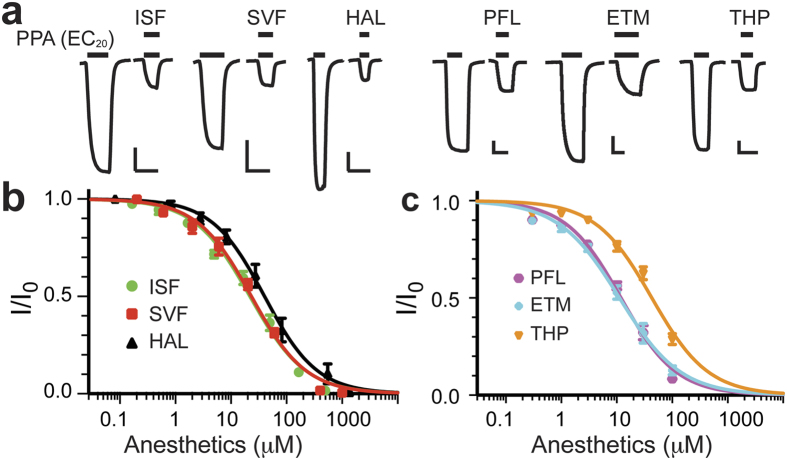
Inhibition of ELIC by general anesthetics. (**a**) Representative ELIC current traces elicited by propylamine (PPA) at EC_20_ in the absence and presence of the general anesthetics isoflurane (ISF, 50 μM), sevoflurane (SVF, 60 μM), halothane (HAL, 82 μM), propofol (PFL, 30 μM), etomidate (ETM, 30 μM), or thiopental (THP, 100 μM). The scale bars are 0.2 μA (vertical) and 30s (horizontal). The data were normalized to the current at EC_20_ in the absence of anesthetics and fit to the Hill equation (solid lines, n ≥ 6) for the volatile (**b**) and intravenous (**c**) general anesthetics. The best-fit parameters for both (**b**,**c**) are provided in Supplementary Table 1. The data are reported as the mean ± SEM and error bars less than the symbol size are not visible.

As a homolog to eukaryotic pLGICs, ELIC shares similar pharmacological profiles of eukaryotic cation-conducting pLGICs with respect to modulation by general anesthetics. Both volatile and intravenous anesthetics inhibited ELIC in a concentration dependent manner (Fig. 1). At the agonist propylamine (PPA) concentration eliciting 20% of the maximal current (EC_20_), isoflurane, sevoflurane, and halothane produced 50% inhibition (IC_50_) at concentrations of 21.9 ± 1.5, 23.2 ± 1.6, and 40.0 ± 4.5 μM, respectively (Fig. 1b and Supplementary Table 1). The intravenous anesthetics propofol, etomidate and thiopental inhibited ELIC with IC_50_ of 11.9 ± 1.1, 11.2 ± 1.1 and 42.8 ± 1.1 μM, respectively (Fig. 1c and Supplementary Table 1). These results are comparable to the corresponding IC_50_ values observed on neuronal nAChRs^31^,^32^,^34^,^35^,^36^,^37^,^38^ (Supplementary Table 1), suggesting that ELIC is a suitable model for understanding anesthetic actions on pLGICs.

### Double occupancy binding of isoflurane inside the ELIC pore

**Figure 2:**
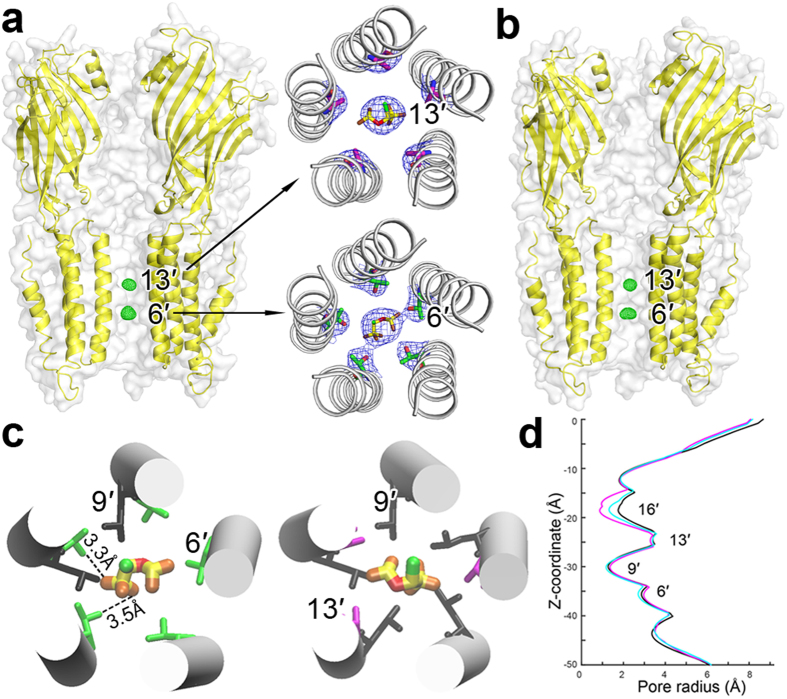
Crystal structures of ELIC with isoflurane bound within the pore. Side views of ELIC bound with two isoflurane molecules near residues T237(6′) and A244(13′) in (**a**) the resting and (**b**) desensitized states. The F_Ο_-F_C_ omitted electron density maps (green) are contoured at 5.0 σ level with a carve distance of 1.8 Å for isoflurane molecules. Two inserted top views in (**a**) show 2F_Ο_-F_C_ electron density maps contoured at 1.0 σ level for isoflurane bound to the 13′ and 6′ positions of ELIC in the resting conformation. (**c**) ELIC residues within 4 Å in contact with isoflurane: T237(6′), green; L240(9′), black; and A244(13′), magenta. Dashlines highlight potential hydrogen bonding between isoflurane and T237(6′). (**d**) Isoflurane binding induced an inward movement of the upper portion of TM2 and reduced pore radius near 16′. The pore profile of apo ELIC (black), ELIC-isoflurane (magenta), ELIC-isoflurane-agonist 3-bromopropylamine (cyan) were calculated using the HOLE program^70^ based on their crystal structures (PDB codes: 3RQU, 4Z90, 4Z91). The z-coordinate is parallel to the channel axis and the zero point of the pore radius is overlapped with the channel axis.

Among the general anesthetics used for functional measurements (Fig. 1), we successfully co-crystalized isoflurane with ELIC in the absence and presence of the agonist 3-bromopropylamine (BrPPA) and determined structures of the complexes up to a 3.0-Å resolution. Crystallographic and refinement parameters are summarized in Table 1. For ELIC crystallized with isoflurane but without an agonist (presumed in the resting state), the F_O_-F_C_ omit electron density map (Fig. 2a) shows strong electron densities for double isoflurane occupancies inside the pore near residues T237(6′) and A244(13′). The numbers in the parentheses are conventional prime notations for pore-lining residues of pLGICs^39^. Nearly equal electron densities observed at the two sites suggest that isoflurane has similar binding affinities to both sites. The 2F_O_-F_C_ electron density maps (inserts of Fig. 2) show refined structures of two isoflurane molecules inside the pore. For ELIC crystallized in the presence of both isoflurane and the agonist BrPPA (presumed in a desensitized state), the F_O_-F_C_ omit electron density map also indicates double isoflurane occupancies in the pore (Fig. 2b). Isoflurane at the 6′ position can form hydrogen bonds with the hydroxyl group of T237 in addition to hydrophobic contacts with L240 at the 9′ position, while isoflurane binding at the 13′ position is dominated by hydrophobic interactions with the side chain of L240 (Fig. 2c).

A structural comparison of the isoflurane-bound ELIC (Fig. 2a) with apo ELIC (PDB code: 3RQU)^40^ revealed an inward movement of the upper part of the TM2 helices and pore contraction near 16′, where the pore radius was reduced by 0.7 Å upon isoflurane binding (Fig. 2d). This conformational change may result from hydrophobic contacts between isoflurane and nearby residues and consequently aids in stabilizing a closed channel structure. Such a conformational change was also observed in the structure of ELIC co-crystallized with isoflurane in the presence of agonists but to a lesser degree (Fig. 2d).

The bromine atom of the agonist BrPPA co-crystalized with ELIC produced bromine-specific anomalous signals in the X-ray diffraction data collected with a wavelength near the K-edge peak of Br (0.9199 Å). The anomalous signals and F_O_-F_C_ electron densities revealed BrPPA binding sites behind loop C and nearby loop A in the extracellular domain (Supplementary Fig. 1). These sites were reported previously to bind agonists, competitive antagonists, and modulators in ELIC^40^,^41^,^42^ and in other pLGICs^43^,^44^.

### Functional relevance of the isoflurane binding sites

**Figure 3:**
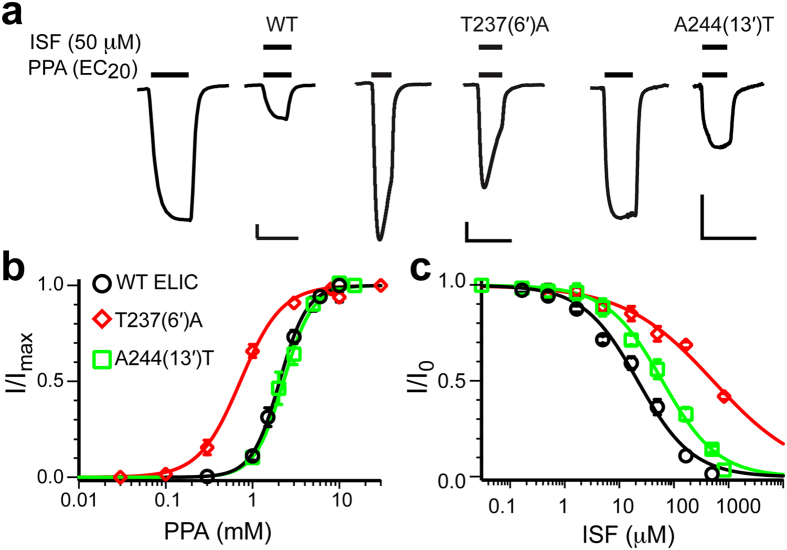
Functional evidence of isoflurane binding to the pore. (**a**) Representative current traces for isoflurane inhibition of WT and two mutants: T237(6′)A; and A244(13′)T. The scale bars are 0.1 μA (vertical) and 30s (horizontal). (**b**) Agonist propylamine (PPA) concentration-response curves for WT and two mutants indicated in (**a**). The data were normalized to the maximum current and fit to the Hill equation (solid lines, n ≥ 5). (**c**) Isoflurane inhibition of WT and the two mutants indicated in (**a**). The data were normalized to the current at EC_20_ in the absence of isoflurane and fit to the Hill equation (solid lines, n ≥ 6). The data are reported as the mean ± SEM and error bars less than the symbol size are not visible.

To establish the functional relevance of the identified isoflurane-binding sites, we made site-directed mutations, one at a time, at positions 6′ (T237A) and 13′ (A244T). Subsequently, we expressed these mutants in *Xenopus laevis* oocytes and measured their functional responses to isoflurane (Fig. 3). The agonist PPA can activate all of the mutant channels and the A244T ELIC mutant has an EC_50_ value nearly identical to the wild-type (WT) ELIC (Fig. 3b). However, these mutant channels, especially T237A, are much less sensitive to isoflurane inhibition than the WT ELIC. Relative to the WT channel (Fig. 1), the IC_50_ of isoflurane increased ∼3-fold for the A244T channel (61.7 ± 5.5 μM) and more than an order of magnitude for the T237A channel (524.8 ± 58.2 μM) (Fig. 3c). The functional consequences of these mutations are associated with changes in the hydrophobicity profile and pore radii near positions 6′ and 13′ (Supplementary Fig. 2). It should be noted that the side chains of the hydrophobic residue at the 9′ position are involved in both of the isoflurane-binding sites (Fig. 2c). The fact that the isoflurane inhibition curve shifts an order of magnitude to the right in the T237A mutant indicates the importance of amphipathic interactions at position 6′ for isoflurane inhibition.

**Figure 4:**
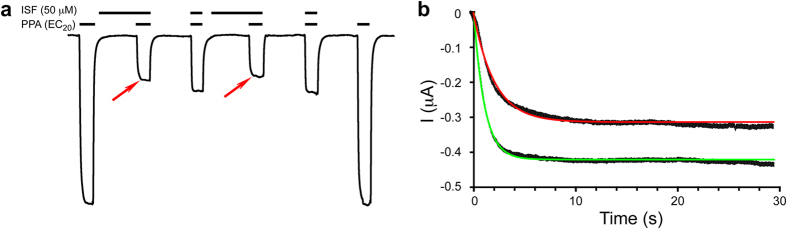
Isoflurane binding stabilized the closed channel. (**a**) A representative trace showing that pre-incubation with isoflurane (red arrows) slowed the onset rate and increased the maximum inhibition. The vertical and horizontal scale bars represent 20 μA and 30 seconds, representatively. (**b**) The onset rate of the channel current can be fitted to a single exponential decay function, I = I_max_ (1−e^(−*t/*^τ)). The fitting results for the onset rate τ and I_max_ for channels pre-incubated with isoflurane (red line) are 1.73 ± 0.04 s and −0.33 ± 0.02 μA, respectively; and 1.25 ± 0.05 s and −0.41 ± 0.03 μA, respectively, for channels without isoflurane pre-incubation (green line). The fitting parameters are expressed as mean ± SEM (n = 15).

Since isoflurane binds to the pore of the resting state of ELIC (Fig. 2a), pre-incubating the oocytes expressing ELIC with isoflurane for a short period of time is expected to enhance the inhibitory effect. As shown in Fig. 4, pre-incubation with isoflurane not only generated greater inhibition, but also decreased the apparent onset rate of agonist-induced current and slowed channel opening. Data indicate that isoflurane binding stabilizes the closed-channel conformation.

### Isoflurane binding characterized by molecular dynamics simulations

Crystal structures show isoflurane binding to ELIC under both resting and desensitized conditions (Fig. 2). In order to determine which functional state of ELIC has more stable isoflurane binding, we performed separate MD simulations of ELIC in a resting conformation as shown in Fig. 2a and a “desensitized” conformation that was obtained from homology modeling on the crystal structure of the desensitized GABA_A_β3 channel^45^. The structure in Fig. 2b was not used for simulations because it is virtually identical to the resting state structure (Fig. 2a), a known crystallization phenomenon reported previously^46^. Compared to the resting ELIC conformation, the upper pore region of the desensitized model is somewhat expanded and the lower pore region is contracted (Supplementary Fig. 3). The differences in pore conformations presented in the resting and “desensitized” ELIC structures used for MD simulations are in good agreement with observations in a recent ^19^F NMR study^47^.

**Figure 5:**
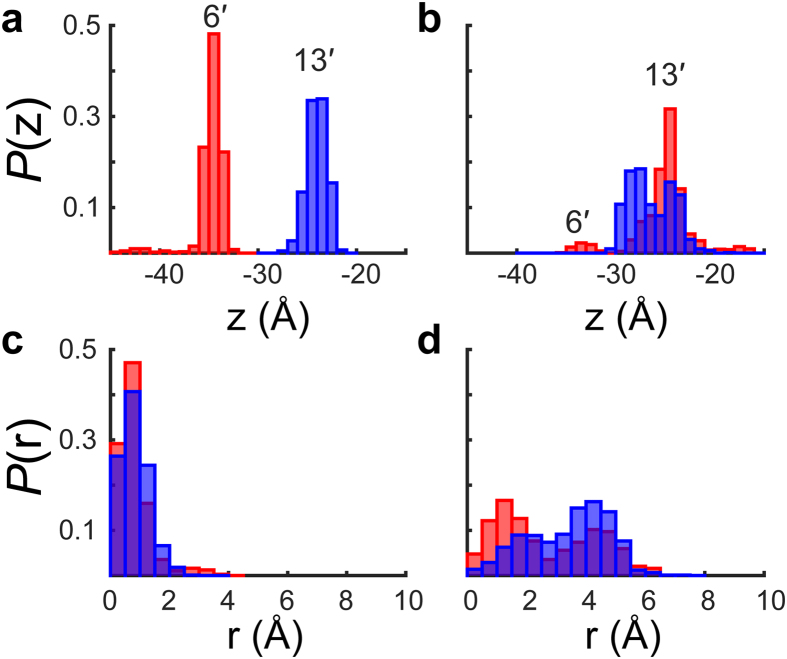
Isoflurane binding is more stable in the closed channel. Probabilities of finding an isoflurane molecule (center of mass) in molecular dynamics (MD) simulations at position z along the pore axis, *P*(z), and at the distance r from the pore axis, *P*(r), were calculated for (**a**,**c)** the closed channel and (**b**,**d**) a desensitized channel. A probability is defined as the ratio of the number of snapshots where isoflurane was found at the position z or at the distance r to the total snapshots chosen from the simulation. The bin widths of 1 Å and 0.5 Å were used for position z and distance r, respectively. Isoflurane motion in each pore conformation was evaluated every 0.1 ns from 100 ns simulations. Color indicates starting positions of isoflurane, 6′ (red) or 13′ (blue), in the MD simulations.

Over the course of the MD simulations of the resting ELIC, isoflurane remained near the same two sites (6′ and 13′) as revealed in the crystal structure (Fig. 5a and Supplementary Fig. 4a). Although isoflurane at the 6′ site transiently migrated to the bottom of the pore at an early stage of the simulation, it quickly returned to the original position. Isoflurane movement perpendicular to the channel axis was largely confined within 2 Å of the pore radius in two replicated simulations (Fig. 5c and Supplementary Fig. 4a), indicating stable binding in the closed pore. In contrast, isoflurane molecules in the modeled desensitized pore moved away substantially from their initial positions (Fig. 5b,d and Supplementary Fig. 4b) and deviated from the center of the pore (Fig. 5d and Supplementary Fig. 4c). Although the modeled pore may not capture quantitative changes of ELIC in the transition from the resting to a desensitized conformation, it at least predicts how expansion at the upper half of the pore affects isoflurane binding.

The binding of isoflurane to the pore may have affected the mobility of the channel. We determined the root mean square fluctuation (RMSF) for the ELIC TMD over the course of the simulations. The RMSF decreased in the closed ELIC bound stably with isoflurane compared to the RMSF of the apo ELIC. In contrast, mobile isoflurane molecules inside the desensitized pore increased the RMSF of the pore region (Supplementary Fig. 5).

## Discussion

### A direct pore-binding mechanism of isoflurane inhibition

The crystal structures of ELIC-isoflurane complex and the functional results of the site-directed mutants point to a direct pore-binding mechanism of isoflurane inhibition. This study has not only revealed the 6′ and 13′ pore positions for the double occupancy of isoflurane in ELIC (Fig. 2), but also demonstrated how pore conformational changes affected isoflurane binding (Fig. 5). The observation of isoflurane in the pore of ELIC under desensitization conditions could be attributed to an open pore-blocking mechanism. By this mechanism, isoflurane occupies and occludes an open pore and then remains in the pore when the channel desensitizes. An open pore-blocking mechanism, however, cannot completely describe the isoflurane inhibition of ELIC since isoflurane also occupies the pore of ELIC in the resting state (Figs. 2 and 4). Furthermore, isoflurane binding to the resting pore stabilizes a closed channel conformation (Figs. 2d and 4, Supplementary Fig. 5). Thus, isoflurane inhibition via a direct pore-binding mechanism is likely accomplished jointly by stabilizing the closed channel conformation and blocking the open channel.

The direct pore-binding mechanism of isoflurane inhibition was also proposed previously based on site-directed mutagenesis studies of nAChRs^26^,^48^ and MD simulations^13^,^14^,^24^ of nAChRs and other cation-conducting pLGICs. Functional studies of WT and mutant muscle-type nAChRs expressed in oocytes and HEK cells suggested that isoflurane binding to the pore was responsible for the inhibition of nAChRs^26^,^48^. MD simulations showed that isoflurane bound to 13′ in the GLIC pore^13^,^14^. Isoflurane binding to 6′ in the GLIC pore was also found in MD simulations, but with a lower probability than to the 13′ position^24^. Interestingly, these simulation results obtained from the open channel GLIC are comparable to our observations of isoflurane binding to the desensitized model of ELIC (Fig. 5).

A remarkable similarity of hydrophobicity profiles in the pore lumens of the cation-conducting channels (Supplementary Fig. 6) may be central to the observation of isoflurane binding to the pore. There is a dichotomy between cation- and anion-conducting channels in their hydrophobic-hydrophilic switching point at position 9′, which has a conserved hydrophobic residue across the entire superfamily. Cation-conducting channels have a hydrophobic upper region of the pore, switching at the 6′ position to a hydrophilic lower pore region. In contrast, the anion-conducting GABA_A_Rs and glycine receptors have hydrophilic residues lining the upper region of their pores. In GLIC, mutating the hydrophobic residue at 13′ to a hydrophilic residue is a critical element to convert the cation-conducting GLIC to an anion-conducting channel^49^. The distinctly different hydrophobicity profile in the pore lumens of the anion-conducting channels may prevent anesthetics from binding to the pore. Indeed, these anion channels are mostly potentiated rather than inhibited by general anesthetics^8^.

### Both pore geometry and hydrophobicity affect isoflurane binding

One may wonder why isoflurane binds specifically to positions 6′ and 13′. Although T237(6′) and A244(13′) have different side-chain volumes (T = 120 Å^3^ vs. A = 90 Å^3^)^50^, the tilting of the TM2 helix makes the pore radii at the 6′ and 13′ positions nearly the same (∼3 Å, Fig. 2d). Such a radius appears necessary to provide an adequate space for isoflurane binding within the pore.

Hydrophobic forces were thought to dominate the interaction of anesthetics with their protein binding sites, as the sensitivity of nAChRs to isoflurane inhibition increased with the pore hydrophobicity^26^. In ELIC, the A244T mutation significantly reduced hydrophobicity^51^ at position 13′ and reduced the channel sensitivity to isoflurane inhibition (Fig. 3). The result seems to be consistent with a dominant contribution of hydrophobic force to anesthetic inhibition. However, our observation of isoflurane at the 6′ position also suggests that amphipathic interactions contribute equally—if not more—to isoflurane binding to the pore. The binding site at 6′ consists of side chains of all five T237(6′) and two L240(9′) residues (Fig. 2c) that form a favorable amphipathic environment to stabilize isoflurane binding through amphipathic interactions. The T237A mutation destroyed the hydrophilic moiety of the amphipathic interaction^52^. In addition, the mutation increased the pore radius at 6′ by ∼1 Å relative to that of the WT ELIC (Supplementary Fig. 2). The pore enlargement at 6′ likely further weakened isoflurane binding, exaggerating the reduction in functional inhibition by isoflurane. Thus, both pore amphiphilicity and geometry can affect anesthetic binding to the pore and alter anesthetic inhibition of channel functions.

### Direct pore binding and allosteric modulation

The structural frameworks presented here and published previously^10^,^53^ support a direct pore-binding mechanism of channel inhibition by anesthetics. A remaining question is how anesthetic molecules reach the pore binding sites. Although a previous MD simulation^14^ suggested that drug molecules dissolved in the membrane could migrate from the interface between two subunits into the pore, in most cases anesthetics may enter the pore from the extracellular pore entrance. For isoflurane (volume, 110 Å^3^), without the conformational flexibility of ELIC as shown in Supplementary Fig. 5, it could not pass through the 16′ and 9′ positions that restrict the pore radius to less than 2 Å (Fig. 2d). Despite the conformational flexibility of the channel, inhibition by direct pore binding is still limited to drugs of a certain size. For example, memantine (volume, 192 Å^3^) could not bind to the pore of ELIC without a mutation to expand the radius of the pore entrance^54^. Thus, for larger anesthetic molecules, a different inhibition mechanism from direct pore binding must apply.

One alternative inhibition mechanism is through allosteric modulation. An increasing number of studies have found that anesthetics have multiple binding sites in a given protein^8^,^10^,^12^,^13^,^14^,^15^,^20^,^21^,^55^. Among the sites reported for anesthetics, allosteric sites predominate. Even though we only observed isoflurane in the pore of the ELIC-isoflurane crystal structures, we do not rule out the possibility that isoflurane also binds to allosteric sites in ELIC. It is not unusual that crystal structures miss some small ligands bound to proteins, especially for low affinity volatile anesthetics like isoflurane. One might argue that any allosteric site will become irrelevant to inhibitory effects once a drug binds to the pore. However, such an argument discounts the possibility that allosteric modulation can affect direct pore binding^18^ or even contribute synergistically with the pore binding inhibitory effect.

## Methods

### Protein expression and purification

The ELIC plasmid was generously provided by Professor Raimund Dutzler’s group at the University of Zürich, Zürich, Switzerland. ELIC was expressed and purified using the protocol reported previously^40^. Briefly, Rosetta (DE3) pLysS (Novagen) cells transformed with the ELIC plasmid were grown in Luria-Bertani media at 37 °C to an OD_600_ of ∼0.9 and transferred to M9 media at 15 °C for ∼2 hours before protein expression was induced with 0.2 mM isopropyl β-D-1-thiogalactopyranoside. Cells were harvested after 24 hours, re-suspended with a buffer containing 150 mM NaCl, 50 mM sodium phosphate at pH 8 and protease inhibitors, and lysed using a M-110Y microfluidizer processor (Microfluidics). The membrane extracts were pelleted and the fusion protein was extracted in the presence of 3.5% (w/v) *n*-Undecyl-β-D-maltoside (Anatrace) followed by purification with a Ni-NTA column (GE Healthcare). The MBP tag was cleaved overnight with HRV3C protease (GE Healthcare) and separated from ELIC using Ni-NTA chromatography. The pentameric ELIC was collected in a buffer containing 10 mM sodium phosphate (pH 8), 150 mM NaCl, 0.025% (w/v) n-dodecyl-β-D-maltoside (Anatrace) by size exclusion chromatography using a Superdex 200 10/300GL column (GE Healthcare). The purified ELIC was concentrated to ∼6 mg/ml for crystallization.

### Crystallography and data analysis

Crystals were obtained using the vapor-diffusion method in sitting drops at 4 °C. ELIC, pre-equilibrated with 0.01–0.02 mg/ml *E. coli* polar lipids (Avanti Polar Lipids), was mixed in 1:1 ratio with the reservoir solution (10–12% PEG 4000, 200 mM ammonium sulfate, and 100 mM MES buffer at pH 6.1). For co-crystalizing ELIC with isoflurane (Penn Veterinary Supply), the reservoir solution was saturated with isoflurane. For co-crystallizing ELIC with 3-bromopropylamine hydrobromide (Sigma-Aldrich), 5 mM 3-bromopropylamine hydrobromide was equilibrated with ELIC for ∼30 minutes before setting up crystallization trays. For cryo-protection, crystals were soaked briefly in the reservoir solution supplemented with 20% glycerol and excess ligands before being flash-frozen in liquid nitrogen.

The X-ray diffraction data were collected on beamline 12–2 at the Stanford Synchrotron Radiation Lightsource (SSRL). The anomalous scattering was acquired near the K-edge peak of Br. The data were indexed, integrated and scaled with the XDS program^56^. All crystals have the P2_1_ space group with two identical pentamers in each asymmetric unit.

A previously published ELIC structure (PDB code: 3RQU, 3.09 Å resolution) was used as a starting template for structure determination of isoflurane-bound ELIC in the absence (3.0 Å) and presence of 3-bromopropylamine (3.4 Å). Two structural regions, including residues 136–156 and residues 285–295, were rebuilt to improve the fitting of electron density. The resulting model was refined iteratively using Phenix^57^. Automatic solvent detection, updating, and refinement were conducted for placing water molecules and followed by manual inspection and adjustment. Isoflurane binding sites were determined based on the F_O_-F_C_ difference map and isoflurane was built inside the pore using Coot^58^. The geometry and stereochemistry of the final model were validated by the program MolProbility^59^. MolProbity scores of 2.01 and 2.12 were obtained for ELIC in the resting and desensitized states, respectively. Initial isoflurane geometry was obtained from the complex structure of apoferritin with isoflurane (PDB code: 1XZ3)^60^. The 3-bromopropylamine binding sites were identified based on the bromine-specific (0.9199 Å) anomalous difference map. Torsional non-crystallographic symmetry (NCS) restraints were applied to all ten subunits of two pentamers in the asymmetric unit. After many cycles of refinement, the final structures were analyzed using Phenix^57^. All molecular graphics were prepared using PyMol^61^ or VMD^62^.

### Electrophysiology

Site-directed mutagenesis of ELIC was introduced with the QuickChange Lightning Kit (Agilent). DNA encoding ELIC and a T7 promoter was inserted into the pCMV-mGFP Cterm S11 NeoKan (Theranostech) vector. Capped complementary RNA was transcribed with the mMessage mMachine T7 kit (Ambion), purified with the RNeasy kit (Qiagen), injected (10–25 ng) into *Xenopus laevis* oocytes (stages 5–6), which were maintained at 18 °C in a modified Barth’s solution^40^. All procedures involving *Xenopus laevis* oocytes were approved by the University of Pittsburgh Institutional Animal Care and Use Committee (IACUC), Protocol 14114745. Two-electrode voltage clamp experiments were performed 16–40 hrs after injection using an Oocyte Clamp OC-725C amplifier (Warner Instruments), Digidata 1440A digitizer (Axon Instruments), and a 20 μl chamber (Automate Scientific). Oocytes were clamped to a holding potential of −40 to −60 mV. The recording solutions contained 130 mM NaCl, 0.1 mM CaCl_2_, 10 mM HEPES, pH 7.0 as well as desired concentrations of agonist and anesthetics. Data were collected and processed using Clampex 10 (Molecular Devices). Non-linear regressions were performed using Prism software (Graphpad).

For measuring anesthetic inhibitory effects, saturated solutions of the volatile anesthetics isoflurane, sevoflurane, and halothane were prepared in the recording solution by stirring overnight at 4 °C. Concentrations of these anesthetics in the recording chamber were calibrated using ^19^F NMR^63^. The intravenous anesthetics, propofol, etomidate, and thiopental, were dissolved directly into the recording solution.

### Molecular dynamics simulations

Four systems were prepared for simulations: (1) the resting ELIC (PDB code: 3RQU)^40^, (2) the resting ELIC-isoflurane (PDB code: 4Z90), (3) the desensitized conformation of ELIC, and (4) the desensitized ELIC bound with isoflurane. The structure of the desensitized ELIC was obtained through homology modeling based on the desensitized structure of GABA_A_Rβ3 (PDB code: 4COF)^45^ using the Modeller program version 9.1.4^64^,^65^. Initial isoflurane positions in system #4 were defined based on the observation in the structure of ELIC co-crystallized with isoflurane and BrPPA (PDB code: 4Z91). ELIC along with the ligands was embedded into an equilibrated membrane of bacterial lipids (1-palmitoyl-2-oleoyl-sn-glycero-3-phosphoethanolamine and 1-palmitoyl-2-oleoyl-sn-glycero-3-phospho-(1′-rac-glycerol) in a 3:1 ratio) in a hexagonal prism, solvated (24,361 TIP3 water molecules), and ionized (62 Na^+^, 23 Cl^−^) using VMD plugins^62^.

NAMD 2.9^66^ and the CHARMM-36 force field^67^ were used for all MD simulations. Each system was energy minimized for 10,000 steps with the ELIC backbone fixed. The system was gradually warmed from 0 to 310 K with a restraint of 1 kcal/mol/Å^2^ on the ELIC backbone. The restraint was gradually removed over 600 ps of further equilibration. Each equilibrated system was simulated for 100 ns at a constant temperature (310 K) and pressure (1 atm) with a 2 fs time step and Langevin damping value of 1 ps^−1^. Particle mesh Ewald was used for long-range electrostatic interactions and a 12 Å cutoff was used for non-bonded interactions. Non-bonded interactions were evaluated at every step, while full electrostatic interactions were evaluated every other step. Periodic boundary conditions were applied to all the simulations. Simulation data were analyzed using VMD^62^, HOLE^68^ and Matlab^69^ programs.

## Additional Information

**Accession Codes**: Coordinates have been deposited to the Protein Data Bank under codes 4Z90 (isoflurane-ELIC complex in the absence of agonists) and 4Z91 (isoflurane-ELIC complex in the presence of agonists).

**How to cite this article**: Chen, Q. *et al.* Direct Pore Binding as a Mechanism for Isoflurane Inhibition of the Pentameric Ligand-gated Ion Channel ELIC. *Sci. Rep.* **5**, 13833; doi: 10.1038/srep13833 (2015).

## Supplementary Material

Supplementary Information

## Figures and Tables

**Table 1:** Data collection and refinement statistics.

	ELIC-ISF^a^	ELIC-ISF-BrPPA^b^
Data collection		
Space group	P2_1_	P2_1_
Cell dimensions		
a, b, c (Å)	105.8, 267.1, 111.1	105.8, 267.6, 111.4
α, β, γ (°)	90, 106.9, 90	90, 107.8, 90
Wavelength (Å)	0.9795	0.9199
Resolution (Å)	39.63-3.00 (3.16-3.00)	40.01-3.39 (3.46-3.39)
Rpim (%)	5.5 (83.9)	6.5 (89.4)
R_merge_ (%)	25.1 (380.1)*	14.6 (196.9)
CC_1/2_	0.998 (0.574)	0.997 (0.445)
<I/σ>	14.9 (1.6)	12.2 (1.0)
No. observations	2512986 (361593)	560028 (28145)
Completeness (%)	99.9 (100)	98.1 (91.6)
Redundancy	21.4 (21.1)	7.0 (6.6)
Wilson B factor (Å^2^)	87.3	106.9
Refinement		
Resolution (Å)	36.97-3.00	34.89-3.39
No. reflections	117183 (3692)	156498 (4560)
R_work_	0.2090 (0.3753)	0.1923 (0.3387)
R_free_	0.2432 (0.3865)	0.2466 (0.4133)
No. atoms		
Protein	25270	25270
Solvent	420	81
Ligand	160	180
B-factors (Å^2^)		
Proteins	97.6	116.3
Solvent	91.6	91.0
ISF	123.5	149.3
Br		222.0
R.m.s deviations		
Bond lengths (Å)	0.014	0.011
Bond angles (°)	1.328	1.430

^a^Merged from five datasets;

^b^single dataset with anomalous scaling.

^*^The larger value is due to the merging of five datasets.
