# Supplementary material for: Direct Pore Binding as a Mechanism for Isoflurane Inhibition of the Pentameric Ligand-gated Ion Channel ELIC
Source: Sci Rep. 2015 Sep 8;5:13833. doi: 10.1038/srep13833 (PMC4561908; doi:10.1038/srep13833)
Supplement: Supplementary Information [file srep13833-s1.pdf]

Direct Pore Binding as a Mechanism for Isoflurane Inhibition of the Pentameric Ligand-gated Ion Channel ELIC

Qiang Chen[1], Monica N. Kinde[1], Palaniappa Arjunan[1,2], Marta M. Wells[1,4], Aina E. Cohen[5], Yan Xu[1,2,3], Pei Tang[1,2,4,*]
[1]Department of Anesthesiology, [2]Department of Pharmacology and Chemical Biology, [3]Department of Structural Biology, [4]Department of Computational and System Biology, University of Pittsburgh School of Medicine; [5]Stanford Synchrotron Radiation Lightsource

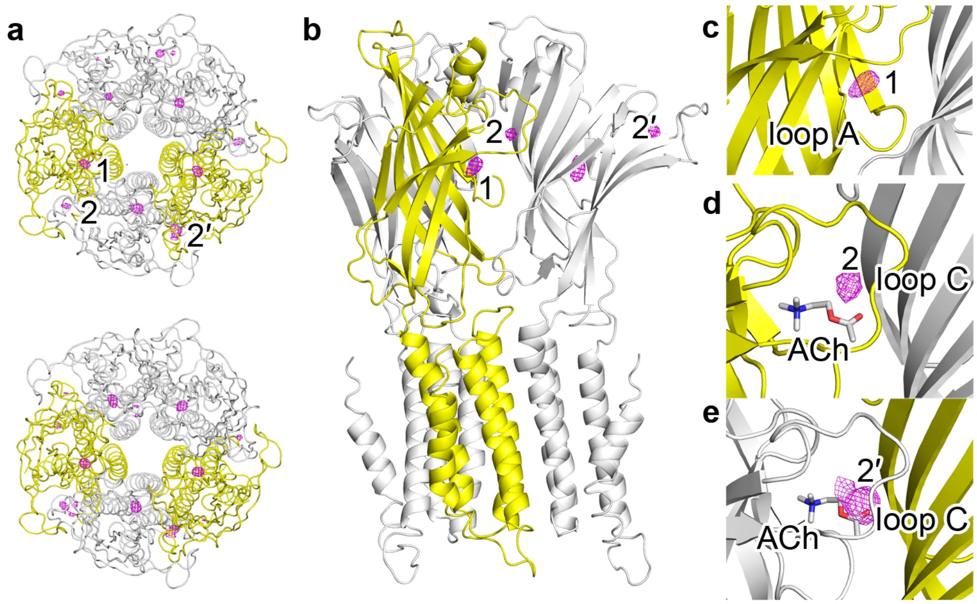

Supplementary Figure 1: The agonist 3-bromopropylamine (BrPPA) binding sites in the extracellular domain of ELIC. (**a**) Top views of two ELIC pentamer structures from an asymmetric unit showing bromine-specific anomalous signals (magenta) in the extracellular domain. Three sets of non-equivalent signals, labeled as 1, 2 and 2', are observable. (**b**) A side view of the BrPPA binding sites in ELIC. For clarity, only three of the five subunits are shown. (**c**) A binding site for BrPPA near loop A, (**d**) a binding site for BrPPA behind loop C and above the acetylcholine (ACh) position identified previously[1], and (**e**) a binding site for BrPPA behind loop C and partially overlapped with the ACh site. All of the bromine-specific anomalous signals were contoured at a 4σ level.

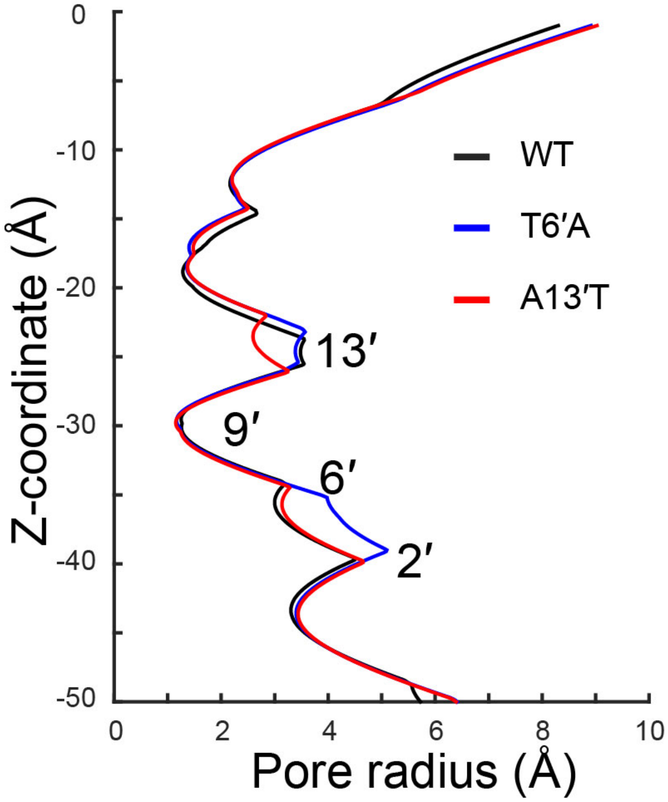

Supplementary Figure 2: Potential changes in pore radius profile introduced by mutations of pore-lining residues. We performed *in silico* mutations A244T and T237A at positions 13′ and 6′, respectively. The A13′T mutation reduced the pore radius by ~1 Å at position 13′ in comparison to the WT ELIC, while the T6′A mutation expanded the pore radius up to ~1 Å around position 6′. *In silico* mutations and subsequent energy minimizations were performed using the programs VMD[2] and NAMD[3]. The pore profiles were calculated using the HOLE program[4].

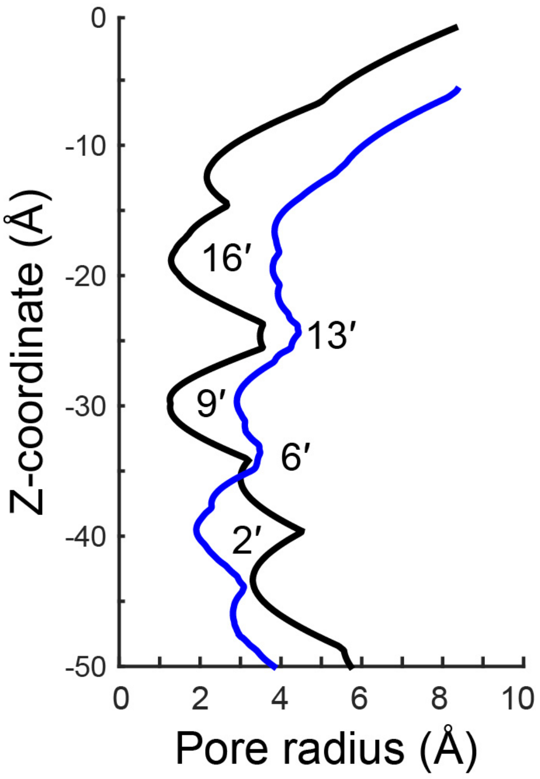

Supplementary Figure 3: The pore profile difference between the modeled desensitized ELIC and the resting ELIC conformations. The structure of the desensitized ELIC was modeled based on the desensitized structure of $GABA_AR\beta3$ (PDB code: 4COF)[5]. The desensitized pore profile (blue) was calculated using the program HOLE and compared with the pore profiles of the resting ELIC (black). As depicted in the desensitized pore, the upper region at 16′ of the pore expanded up to 2.5 Å and the lower region near 2′ of the pore contracted up to 2.5 Å.

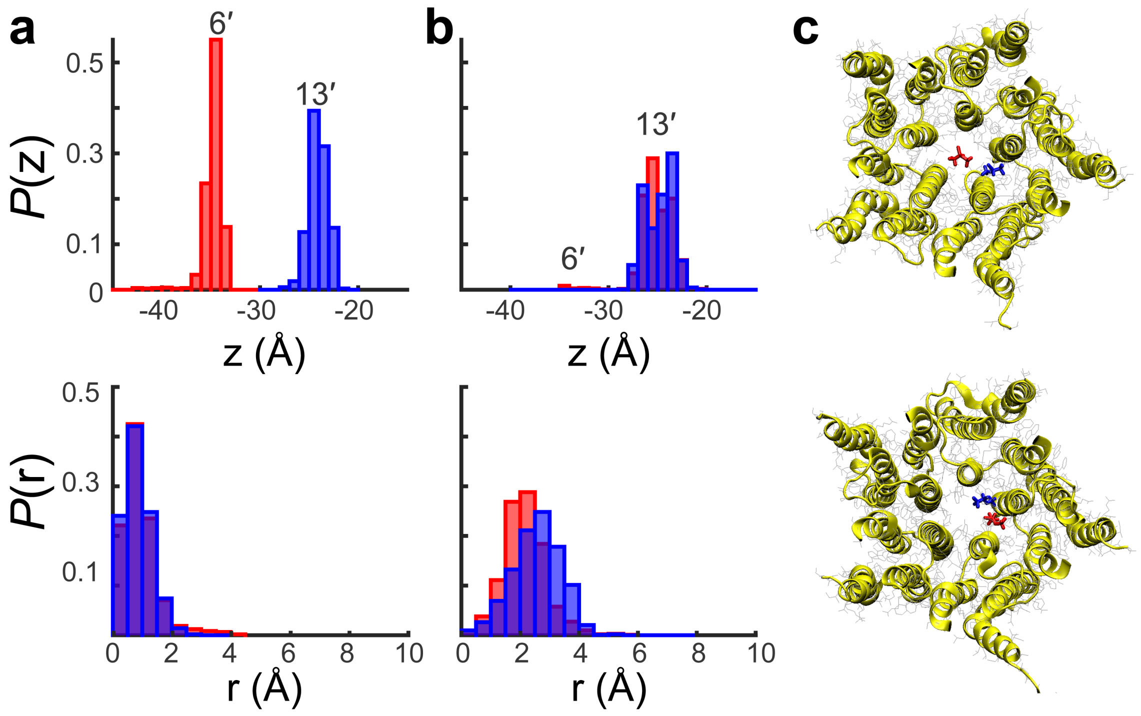

Supplementary Figure 4: Replicated molecular dynamics (MD) simulations of isoflurane binding to ELIC in the resting (**a**) and (**b**) a desensitized pore conformation**.** The results in (a) and (b) are consistent with the data presented in Fig. 5. Isoflurane motion in each pore conformation was evaluated every 0.1 ns from 100 ns simulations. Color indicates starting positions of isoflurane, 6′ (red) or 13′ (blue), in the MD simulations. In the resting state, isoflurane motion was confined to the sites 13′ or 6′ along the pore axis and within 2 Å of the pore axis. However, in a desensitized pore conformation, both isoflurane molecules were more populated near 13′ and moved more broadly radially. (**c**) Representative snapshots from MD simulations showing that isoflurane deviated from the pore axis in the desensitized ELIC. One of the isoflurane molecules (upper panel) migrated away from the center of the pore and entered a site between two TM2 helices. Both isoflurane molecules in the bottom panel moved away from the center of the pore.

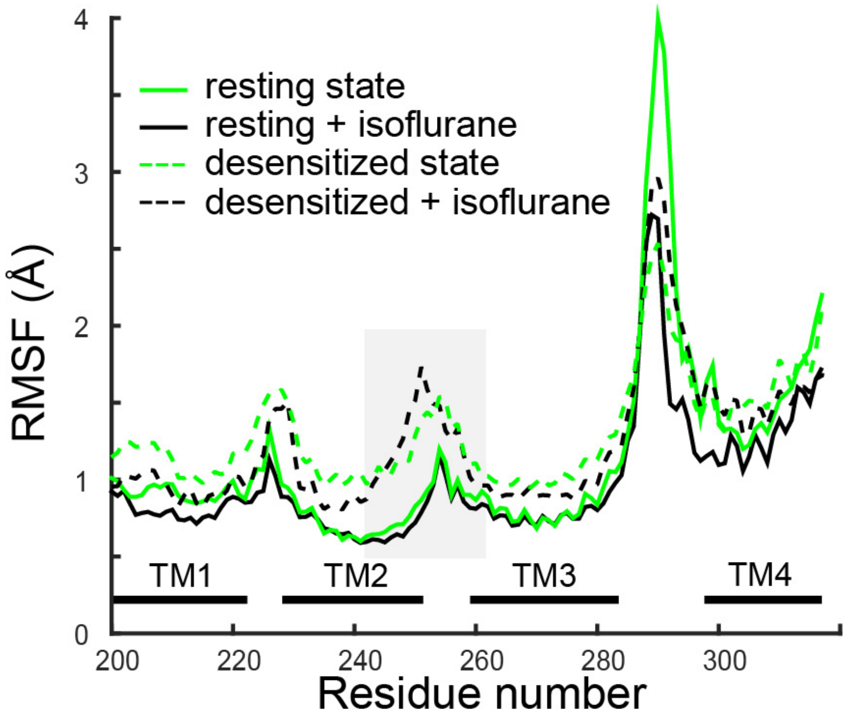

Supplementary Figure 5: Root Mean Square Fluctuation (RMSF) of the ELIC transmembrane domain (TMD) in four simulation systems: (i) the resting ELIC without isoflurane (green); (ii) the resting ELIC with two isoflurane molecules bound to the pore (black); (iii) the desensitized ELIC without isoflurane (green dash); and (iv) the desensitized ELIC with two isoflurane molecules bound to the pore (black dash). Two replicate simulations were ran for each system, each simulation lasting for 100 ns. The RMSF for each system was the average result from its replicate simulations. Note that the RMSF values of the pore region (TM2) are much higher in the desensitized ELIC than in the resting ELIC. Moreover, isoflurane binding reduced the flexibility of the upper pore region (highlighted in gray) in the resting ELIC but increased the flexibility of the same region in the desensitized ELIC.

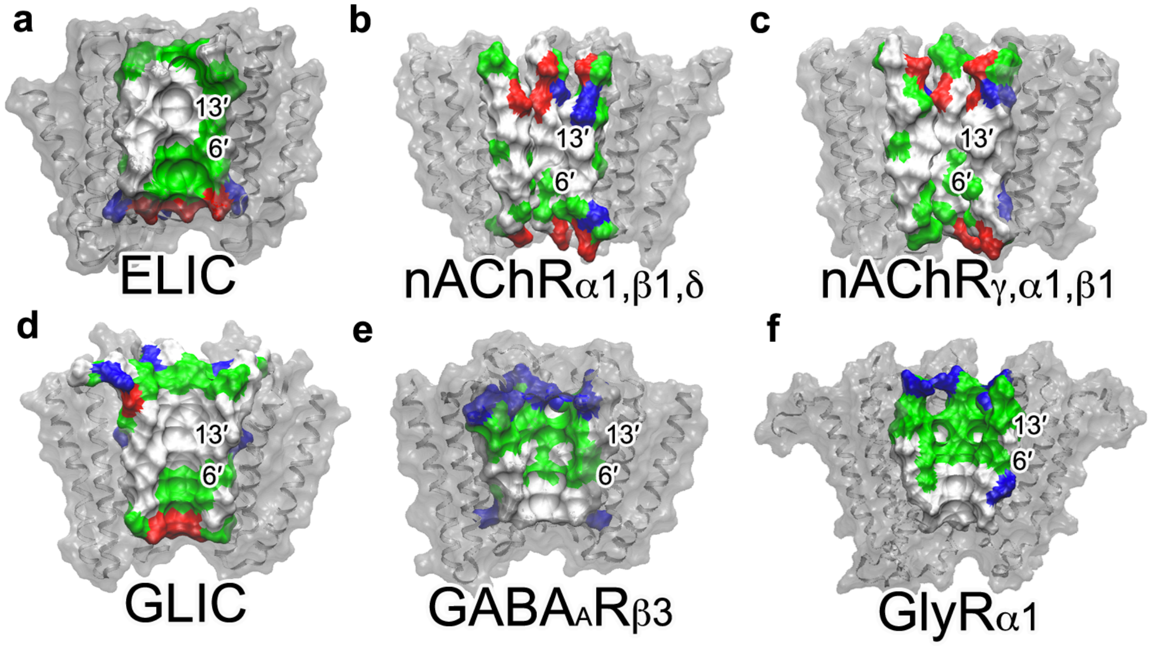

g

TM2

```
   ELIC 228 FSERLQTSFTLMLTVVAYAFYTSNILPRLPY 258
nAChRα1 239 SGEKMTLSISVLLSLTVFLLVIVELIPSTSS 269
     β1 245 AGEKMSLSISALLALTVFLLLLADKVPETSL 275
      δ 253 SGEKMSTAICVLLAQAVFLLLTSQRLPETAL 283
      γ 247 GGQKCTLSISVLLAQTIFLFLIAQKVPETSL 277
   GLIC 221 YEANVTLVVSTLIAHIAFNILVETNLPKTPY 251
GABAARβ3 247 SAARVALGITTVLTMTTINTHLRETLPKIPY 277
 GlyRα1 249 APARVGLGITTVLTLTTQSSGSRASLPKVSY 279
              -2′  2′  6′ 9′  13′16′ 20′
```

Supplementary Figure 6: Hydrophobicity profiles of the pore lumens of selected pentameric ligand-gated ion channels: (**a**) ELIC (PDB code: 3RQU), (**a**) α1, β1, and δ subunits of nAChR (PDB code: 2BG9), (**c**) γ, α1, and β1 subunits of nAChR (PDB code: 2BG9), (**d**) GLIC (PDB code: 3EAM), (**e**) $GABA_AR\beta 3$ (PDB code: 4COF), and (**f**) GlyRα1 (PDB code: 2M6I). For clarity, only three subunits are displayed for each channel. The surfaces of the pore lumens are colored based on the residue type: negatively charged (red), positively charged (blue), polar (green), nonpolar (white). Clearly, all of the cation-conducting channels shown in (**a**)-(**d**) have hydrophobic lumens above the 6′ position and hydrophilic lumens below the 6′ position. In contrast, the anion-conducting channels in (**e**) and (**f**) have an inverted hydrophobicity distribution along the pore lumen. (**g**) The sequence alignment of the second transmembrane domain (TM2) of the selected proteins, for which the pore-lining residues are marked.

Supplementary Table 1: Inhibition of representative general anesthetics on ELIC and nAChRs.

Anesthetic	IC_{50} for ELIC (μM)‡	IC_{50} for nAChRs (μM)	Clinical EC_{50}* (μM)
Isoflurane	21.9 ± 1.5	α4β2 (chicken): 85[6]<br>α4β2 (rat): 34[7]<br>α4β2 (human): 82;<br>α2β4: 25; α3β4: 56;[8]	280[9,10]
Sevoflurane	23.2 ± 1.6	α4β2 (rat): 98[7]	330[9,10]
Halothane	40.0 ± 4.5	α4β2 (human): 27<br>α2β4 (human): 47<br>α4β4 (human): 105[7]	230[9,10]
Propofol	11.9 ± 0.8	α4β2 (chicken): 19[6]<br>α4β2 (rat): 4.5[7]	1.5[10]
Etomidate	11.2 ± 0.9	α4β4 (chicken): 33[11]<br>α4β2 (rat): 49[12]<br>α7 (chicken): 25.3[12]	2, 2.4, 4, 8.7[11,13,14]
Thiopental	42.8 ± 3.3	α4β4 (chicken): 84[11]<br>α4β2 (rat): 26[15]<br>α7 (human): 40.6[16]	25[9,10]

‡IC_{50} values are expressed as mean ± SEM.

*Anesthetizing concentrations of these drugs in animals.

References

1. Pan, J. et al. Structure of the pentameric ligand-gated ion channel ELIC cocrystallized with its competitive antagonist acetylcholine. *Nat. Commun.* **3**, 714 (2012).
2. Humphrey, W., Dalke, A. & Schulten, K. VMD: visual molecular dynamics. *J. Mol. Graph.* **14**, 33-8, 27-8 (1996).
3. Phillips, J.C. et al. Scalable molecular dynamics with NAMD. *J Comput Chem* **26**, 1781-802 (2005).
4. Smart, O.S., Goodfellow, J.M. & Wallace, B.A. The pore dimensions of gramicidin A. *Biophys. J.* **65**, 2455-60 (1993).
5. Miller, P.S. & Aricescu, A.R. Crystal structure of a human GABAA receptor. *Nature* **512**, 270-5 (2014).
6. Flood, P., Ramirez-Latorre, J. & Role, L. Alpha 4 beta 2 neuronal nicotinic acetylcholine receptors in the central nervous system are inhibited by isoflurane and propofol, but alpha 7-type nicotinic acetylcholine receptors are unaffected. *Anesthesiology* **86**, 859-65 (1997).
7. Violet, J.M., Downie, D.L., Nakisa, R.C., Lieb, W.R. & Franks, N.P. Differential sensitivities of mammalian neuronal and muscle nicotinic acetylcholine receptors to general anesthetics. *Anesthesiology* **86**, 866-74 (1997).
8. Cardoso, R.A., Yamakura, T., Brozowski, S.J., Chavez-Noriega, L.E. & Harris, R.A. Human neuronal nicotinic acetylcholine receptors expressed in Xenopus oocytes predict efficacy of halogenated compounds that disobey the Meyer-Overton rule. *Anesthesiology* **91**, 1370-7 (1999).
9. Franks, N.P. & Lieb, W.R. Molecular and cellular mechanisms of general anaesthesia. *Nature* **367**, 607-614 (1994).
10. Franks, N.P. & Lieb, W.R. Which molecular targets are most relevant to general anaesthesia? *Toxicol. Lett.* **100-101**, 1-8 (1998).
11. Flood, P. & Krasowski, M.D. Intravenous anesthetics differentially modulate ligand-gated ion channels. *Anesthesiology* **92**, 1418-25 (2000).
12. Belelli, D. et al. The in vitro and in vivo enantioselectivity of etomidate implicates the GABAA receptor in general anaesthesia. *Neuropharmacology* **45**, 57-71 (2003).
13. Krasowski, M.D. & Harrison, N.L. General anaesthetic actions on ligand-gated ion channels. *Cell. Mol. Life Sci.* **55**, 1278-303 (1999).
14. Giese, J.L. & Stanley, T.H. Etomidate: a new intravenous anesthetic induction agent. *Pharmacotherapy* **3**, 251-8 (1983).
15. Downie, D.L., Franks, N.P. & Lieb, W.R. Effects of thiopental and its optical isomers on nicotinic acetylcholine receptors. *Anesthesiology* **93**, 774-83 (2000).
16. Coates, K.M., Mather, L.E., Johnson, R. & Flood, P. Thiopental is a competitive inhibitor at the human alpha7 nicotinic acetylcholine receptor. *Anesth. Analg.* **92**, 930-3 (2001).
